# EHMTI-0105. CGRP and VIP levels as predictors of efficacy of onabotulinumtoxin type A in chronic migraine

**DOI:** 10.1186/1129-2377-15-S1-E24

**Published:** 2014-09-18

**Authors:** E Cernuda-Morollón, P Martínez-Camblor, E Serrano-Pertierra, D Larrosa, C Ramón, J Pascual

**Affiliations:** 1 Neurology, Hospital Universitario Central de Asturias and Ineuropa, Oviedo, Spain; 2 OIB, Oficina de Investigación Sanitaria, Oviedo, Spain

## Introduction

The mechanism of action of Onabotulinumtoxin type A (onabotA) in chronic migraine (CM) is unknown.

## Aim

To analyse a potential relationship between CGRP and VIP levels and onabotA response.

## Methods

CGRP and VIP levels were determined in antecubital vein samples by ELISA outside a migraine attack prior to treatment with onabotA. OnabotA was administered according to PREEMPT at least twice. A patient was considered as a moderate responder when both: 1) moderate-severe headaches were reduced by between 33-66%; and 2) benefit in a visual scale of 0-100 was recorded between 33-66%. Patients were considered as excellent responders when both items improved > 66%. Those without improvement of at least one-third in the two items were considered as nonresponders.

## Results

We assessed samples from 81 CM patients and 33 controls. CGRP and VIP levels were significantly increased in CM population. CGRP and, to a lesser degree, VIP levels were significantly increased in responders vs nonresponders. For CGRP, a threshold of 72 pg/ml positively correlated with 95% of nonresponders. The probability of being a responder to onabotA was 28 times higher with a CGRP level above the threshold of 72 pg/ml. Even though the sensitivity for the calculated threshold for VIP was poor, the probability that CM patients with low CGRP levels will respond to onabotA was significantly higher in those patients with high VIP levels.

## Conclusions

Interictal CGRP and, to a lesser degree, VIP levels are of help on predicting onabotA response. Supported by Pi11/00889 FISSS and Allergan-Eurasia MAF/ISS/NS/CM/003 grants.

Conflict of interest.

